# Interface residues of transient protein-protein complexes have extensive intra-protein interactions apart from inter-protein interactions

**DOI:** 10.1186/s13062-019-0232-2

**Published:** 2019-01-15

**Authors:** Srinivasan Jayashree, Pavalam Murugavel, Ramanathan Sowdhamini, Narayanaswamy Srinivasan

**Affiliations:** 1 School of Bioscience & Technology, Vellore Institute of Technology, VIT University, Vellore, 632014 India; 2 National Centre for Biological Sciences, TIFR, UAS-GKVK Campus, Bellary road, Bangalore, 560065 India; 3 Molecular Biophysics Unit, Indian Institute of Science, Bangalore, 560012 India

**Keywords:** Interfacial residues, Molecular interactions, Molecular recognition, Protein interactions, Protein-protein complexes, Protein-protein interfaces

## Abstract

**Background:**

Protein-protein interactions are crucial for normal biological processes and to regulate cellular reactions that affect gene expression and function. Several previous studies have emphasized the roles of residues at the interface of protein-protein complexes in conferring stability and specificity to the complex. Interface residues in a protein are well known for their interactions with sidechain and main chain atoms with the interacting protein. However, the extent of intra-protein interactions involving interface residues in a protein-protein complex and their relative contribution in comparison to inter-protein interactions are not clearly understood. This paper probes this feature using a dataset of protein-protein complexes of known 3-D structure.

**Results:**

We have analysed a dataset of 45 transient protein-protein complex structures with at least one of the interacting proteins with a known structure available also in the unbound form. We observe that a large proportion of interface residues (1608 out of 2137 interface residues, 75%) are involved in intra and inter-protein interactions simultaneously. The amino acid propensities of such interfacial residues involved in bifurcated interactions are found to be highly similar to the general propensities to occur at protein-protein interfaces. Finally, we observe that a majority (83%) of intra-protein interactions of interface residues with bifurcated interactions, are also observed in the protein uncomplexed form.

**Conclusions:**

We have shown, to the best of our knowledge for the first time, that a vast majority of the protein-protein interface residues are involved in extensive intra-protein interactions apart from inter-protein interactions. For a majority of such interface residues the microenvironment in the tertiary structure is pre-formed and retained upon complex formation with its cognate partner during transient interactions.

**Reviewers:**

This article was reviewed by Arumay Pal and Mallur Madhusudhan.

**Electronic supplementary material:**

The online version of this article (10.1186/s13062-019-0232-2) contains supplementary material, which is available to authorized users.

## Background

Association between two or more proteins is central to many cellular processes [1]. These associations are highly specific both in terms of partnerships between proteins and the three-dimensional (3-D) orientation of the proteins in the associated form [2]. Further, many of these complexes are transient in nature. i.e., the association and disassociation are continuous processes.

In transient protein-protein complexes, the strength of association between proteins is also an important feature that must be maintained at precise levels depending upon the proteins involved and desired time of association between proteins before they disassociate [3]. Indeed, some of the interfacial residues contributing substantial energy of stabilization of the complex are referred as “hot spots”. Mutation of such residues is known to compromise on the binding affinity between the proteins involved [4–6].

Obviously, residues at the protein-protein interface play a crucial role in conferring right level of stability of the complex, as well as in conferring specificity for the association between proteins and their precise 3-D structure. Mutation of such residues can compromise on the stability and/or specificity of the proteins concerned and their complex leading to disease states [7] and other altered properties [8] . What is the role of the interfacial residues in the transient protein-protein complexes when the proteins are in the disassociated form? A detailed analysis shows that a sub-set of interfacial residues with limited mobility act as anchors, thereby contributing to the specificity of association between proteins [9].

However, it is currently not clear, what is the contribution of the interfacial residues in their interactions within the protein? For example, an interfacial residue, like Arginine, may form simultaneous hydrogen bonds within the protein and with the associated protein.

The present work analyzes known 3-D structures of protein-protein complexes, with a view to understand the extent of interaction of interfacial residues within the protein (intra-protein interactions), apart from interacting with residues in the interface of associated protein (inter-protein interactions).

## Methods

### Dataset

According to the earlier literature, at least 176 transient protein-protein complexes are known to have structural information available in both bound and unbound forms [9, 10]. For the current analysis, we imposed a condition that the complex structure should be available in high resolution (equal to or better than 2 Å), with at least one of the two proteins in every complex should have its structure available in the free form. This resulted in 45 Protein Data Bank (PDB - [11]) entries, pertaining to 114 protein chains, of protein-protein complexes of known structure with at least one of the protein structures in a complex, also available in unbound form. We used this condition as we wanted to explore extent of retention of intra-protein interactions, involving interfacial residues, in the bound and free forms. As some of the 45 PDB entries correspond to more than one copy of a protein-protein complex in the crystallographic asymmetric unit, the number of chains in the data set is more than double the number of PDB entries. In such cases, some differences in the structural features were noticed between the copies of the complexes in the asymmetric unit. Therefore, all the 114 chains have been used in the current analysis.

### Identification and categorization of residue-residue interactions

A pair of atoms, one from each of the two proteins in a protein-protein complex, that are involved in interactions are considered as interfacial atoms. Interacting atoms were identified using PIC server [12] (see below). If an interfacial atom in the complex is from the sidechain of a residue then the residue is considered as an interfacial residue. A collection of interfacial residues in a protein of the complex is considered as the interfacial region of that protein.

Both inter-protein and intra-protein interactions were identified using PIC server [12]. If an interaction involves at least one side chain atom of a residue, then that residue is considered to be involved in interaction and the interaction is included for further analysis. All side chains in a protein which are involved in interaction with side chain or main chain of the bound protein are considered as protein-protein interfacial residues. Interactions were distinguished and listed according to types (such as van der Waals, hydrogen bond, aromatic-aromatic and salt bridge) across the bound proteins. It is possible that the same residue pair could be listed in more than one type if there are simultaneous van der Waals and hydrogen bond interactions etc. In such instances, the pair with interacting residues was counted only once, though the number of interactions between the same two residues could be more than one. It is also possible that the same residue is interacting with more than one residue, within or across subunits. Such residue-residue interactions were counted separately.

### Amino acid propensity calculations

The propensities of amino acids to be present at the interface and engaged in bifurcated interactions (both intra- and inter-protein) were measured as per the standard Chou-Fasman [13] type propensity calculations. These two interactions could be inferred through results from PIC server, by two separate runs (one with option for ‘protein-protein interactions’ and another with option for ‘intra-protein interactions’).

### Calculation of interaction energy

PPCheck was used to identify and quantify interactions in protein-protein interfaces [14]. Residues within 10 Å of C^α^ -C^α^ distance are considered and energies were calculated considering the nature of interaction. Energy is measured as the sum of van der Waals, electrostatics and hydrogen bond interactions. The energy contributions of these types of interactions are as per enthalpic calculations. Additionally, a distance-dependent dielectric has been used and hydrogen bonds are analysed after fixing hydrogen atoms.

In order to measure the strength of interactions harboured by interface residues which are involved in bifurcated interactions, intra-protein interactions were initially identified using the ‘intra-protein interaction’ option in PIC server. For each interface residue with bifurcated interactions, the micro-environments of all interacting intra-protein residues were alone calculated for PPCheck calculations for intra-protein interactions. Where multiple interactions are observed between two residues, the total energy of all the interactions between the residues is associated with the residue pair.

## Results and discussion

### Extent of intra-protein interactions by protein-protein interfacial residues

In the current analysis, we have used a dataset of 45 protein-protein complexes of known crystal structure (determined at 2 Å or better resolution) with 3-D structure of at least one of the proteins in every complex available in the uncomplexed form (Table 1, Additional file 1: Table S1). We used the uncomplexed protein structures to explore the extent of retention of intra-protein interactions involving interfacial residues in the form complexed to another protein.

**Table 1:** Transient protein-protein complexes of known 3-D structure employed for the analysis. For every entry in this dataset, a corresponding PDB entry is observed in the ‘unbound’ for at least one of the two proteins in the complex (details are in Additional file 1: Table S1)

PDB code	Resolution (Å)	Complex structure title	Source organism
1AY7	1.7	Ribonuclease SA complex with Barstar	*Bacillus amyloliquefaciens*
1B0N	1.9	SINR/SINI protein complex	*Bacillus subtilis*
1F3V	2	N-terminal domain of TRADD and the TRAF domain of TRAF2	*Homo sapiens*
1FM0	1.45	Molybdopterin Synthase (MOAD/MOAE)	*Escherichia coli*
1G73	2	SMAC bound to XIAP-BIR3 domain	*Homo sapiens*
1GL4	2	Nidogen-1 G2/Perlecan IG3 Complex	*Mus musculus*
1HX1	1.9	BAG domain in complex with the HSC70 ATPASE domain	*Bos taurus*
1JIW	1.74	APR-APRin complex	*Pseudomonas aeruginosa*
1NRJ	1.7	Signal Recognition Particle Receptor Beta-Subunit in Complex with the SRX Domain from the Alpha-Subunit	*Saccharomyces cerevisiae*
1O6S	1.8	Internalin (*Listeria monocytogenes*) / E-Cadherin (human) Recognition Complex	*Homo sapiens*
1OR7	2	Escherichia coli sigmaE with the Cytoplasmic Domain of its Anti-sigma RseA	*Escherichia coli*
1PXV	1.8	Staphostatin-staphopain complex	*Staphylococcus aureus*
1R0R	1.1	Protein Inhibitor, OMTKY3, and the Serine Protease, Subtilisin Carlsberg	*Bacillus licheniformis*
1T0F	1.85	TnsA/TnsC(504–555) complex	*Escherichia coli*
1T6G	1.8	*Triticum aestivum* xylanase inhibitor-I in complex with aspergillus niger xylanase-I	*Aspergillus niger*
1TA3	1.7	Crystal Structure of xylanase (GH10) in complex with inhibitor (XIP)	*Aspergillus nidulans*
1UUZ	1.8	IVY:A NEW FAMILY OF PROTEIN	*Gallus gallus*
1WQJ	1.6	Insulin-Like Growth Factors (IGFs) in complex with IGF Binding Proteins (IGFBPs)	*Homo sapiens*
1Z0J	1.32	Structure of GTP-Bound Rab22Q64L GTPase in complex with the minimal Rab binding domain of Rabenosyn-5	*Homo sapiens*
1Z5Y	1.94	N-Terminal Domain Of The Electron Transfer Catalyst DsbD and The Cytochrome c Biogenesis Protein CcmG	*Escherichia coli*
1ZHH	1.94	Apo Form of Vibrio Harveyi LUXP Complexed with the Periplasmic Domain of LUXQ	*Vibrio harveyi*
1ZLH	1.7	Crystal structure of the tick carboxypeptidase inhibitor in complex with bovine carboxypeptidase A	*Bos taurus*
2APO	1.95	Methanococcus jannaschii Cbf5 Nop10 Complex	*Methanocaldococcus jannaschii*
2CIO	1.5	Papain complexed with fragments of the Trypanosoma brucei cysteine protease inhibitor ICP.	*Carica papaya*
2DFX	1.9	Carboxy terminal domain of colicin E5 complexed with its inhibitor	*Escherichia coli*
2EJF	2	Biotin Protein Ligase (Mutations R48A and K111A) and Biotin Carboxyl Carrier Protein Complex	*Pyrococcus horikoshii*
2ES4	1.85	Lipase-specific foldase in complex with its cognate lipase	*Burkholderia glumae*
2FCW	1.26	LDL Receptor Ligand-Binding Modules 3–4 and the Receptor Associated Protein (RAP).	*Homo sapiens*
2HQS	1.5	TolB/Pal complex	*Escherichia coli*
2J9U	2	ESCRT-I Vps28 C-terminus in complex with the NZF-N domain from ESCRT-II	*Saccharomyces cerevisiae*
2UUY	1.15	Structure of a tick tryptase inhibitor in complex with bovine trypsin	*Bos taurus*
2UYZ	1.4	Non-covalent complex between Ubc9 and SUMO1	*Homo sapiens*
2VN5	1.9	Dockerin-cohesin complex	*[Clostridium] cellulolyticum*
2WEL	1.9	SU6656-bound calcium/calmodulin-dependent protein kinase II delta in complex with calmodulin	*Homo sapiens*
2XGY	1.8	Complex of Rabbit Endogenous Lentivirus (RELIK)Capsid with Cyclophilin A	*Homo sapiens*
2Z0D	1.9	Human Atg4B- LC3(1–120) complex	*Homo sapiens*
3A8I	1.99	Crystal Structure of ET-EHred-5-CH3-THF complex	*Escherichia coli*
3EGV	1.75	Ribosomal protein L11 methyltransferase (PrmA) in complex with trimethylated ribosomal protein L11	*Thermus thermophilus*
3K2M	1.75	Monobody HA4/Abl1 SH2 Domain Complex	*Homo sapiens*
3MN5	1.5	Actin-bound WH2 domains of Spire	*Drosophila melanogaster*
4CJ2	1.5	HEWL in complex with affitin H4	*Gallus gallus*
4DEX	2	Voltage Dependent Calcium Channel beta-2 Subunit in Complex With The CaV2.2 I-II Linker.	*Oryctolagus cuniculus*
4M3K	1.48	Single domain camelid antibody fragment cAb-H7S in complex with the BlaP beta-lactamase	*Bacillus licheniformis*
4MRT	2	Phosphopantetheine Transferase Sfp in Complex with Coenzyme A and a Peptidyl Carrier Protein	*Bacillus subtilis*
4Q57	1.8	plectin 1a actin-binding domain/N-terminal domain of calmodulin complex	*Homo sapiens*

In the complex structures, interactions such as hydrogen bond, van der Waals and salt bridge within the protein and between the bound proteins were identified using PIC [12]. These residues at the protein-protein interface have been analyzed for their intra and inter-protein interactions and the energies associated with the interactions have been calculated. For every interfacial residue, two categories of interactions were considered: (1) interaction made by the residue with the bound protein and (2) simultaneous interaction of the sidechain with the bound protein as well as within the same protein. The first category corresponds solely to interaction across the protein-protein interface. Second category refers to the two kinds of interactions made by a given side chain; interaction made by the residue with a sidechain or main chain atom in the same protein chain and interaction across the protein-protein interface. These two categories of interactions are henceforth referred as “solely inter-protein” and “simultaneous inter and intra-protein interactions” respectively.

Of the 2137 interfacial residues in the entire dataset of 45 complex structures, 529 residues form solely inter-protein interactions. Interestingly, a majority of 1608 interfacial residues form simultaneous inter and intra-protein interactions (Additional file 2: Table S2). Therefore, intra-protein interactions, involving sidechain of interfacial residues, also contribute towards structure and stability of protein-protein complexes. The distribution of percentage residues involved in bifurcated interactions are shown in Additional file 3: Figure S1. Interface residues in most PDB chains are engaged in bifurcated interactions, except five chains (corresponding to PDB entries 2cio, 1f3v, 2uyz, 1gl4 and 2fcw; Additional file 2: Table S2). Out of these, two protein chains (corresponding to PDB codes 1f3v and 2fcw), are shown to undergo large conformational changes in comparison to the unbound form (please see later). In two others (PDB code 1uyz and 1gl4), the other chain is primarily involved in contributing to bifurcated interactions.

The remaining 25% of the interfacial residues show no clear intra-protein interactions, but are involved in inter-protein interactions. The list of residue types involved in this set is almost same as the list of residue types involved in simultaneous inter and intra-protein interactions (see the propensity calculation results below) except for Gln and Lys. While the tendency of Lys not to be involved in intra-protein interaction could be due tothe localized nature of -NH_2_ group at the end of its sidechain, it is not clear why Gln shows higher tendency for inter-protein interactions, than simultaneous intra- and inter-protein interactions.

### Propensities of residue types to form simultaneous intra and inter-protein interactions

**Fig. 1:**
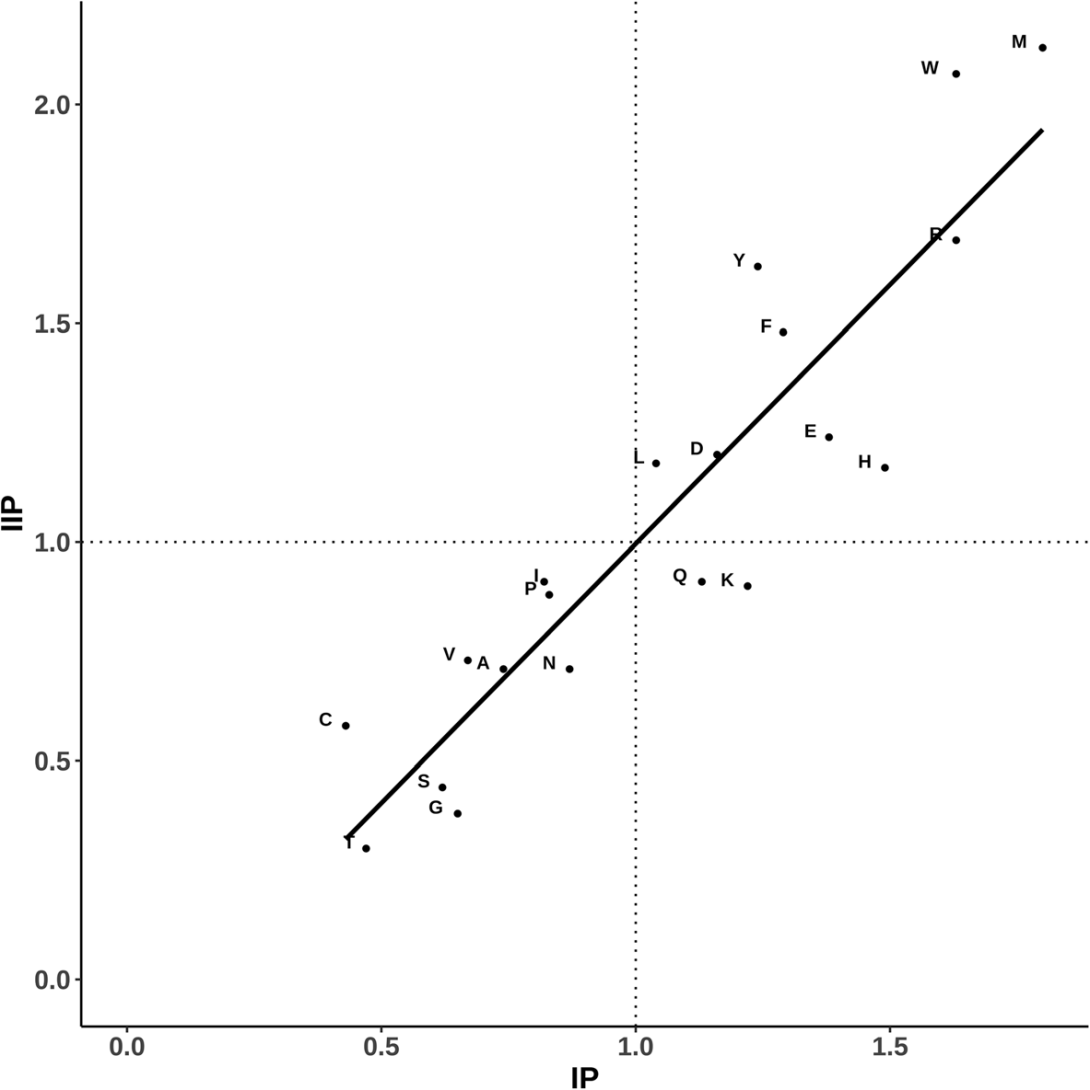
Scatter plot showing the propensities of the residue types to occur in the protein-protein interfaces (IP, along the X-axis) and propensities to form simultaneous inter and intra-protein interactions (IIP, along the Y-axis). Amino acid residues are marked in single letter code. The vertical and horizontal lines at propensity value of 1 are shown in dotted lines. Least-squares fit line is shown. Correlation-coefficient is 0.91

Propensities of each of the 20 residue types to occur in the interface have been reported in several previous publications. In this work, propensities of each of the 20 residue types to form simultaneous inter and intra-protein interactions have been calculated. In order to maintain the consistency, propensities to occur in the interface have also been calculated to facilitate a convenient comparison (Fig. 1). All the residue types, except Gln and Lys, have propensity greater than 1 to form bifurcated interactions (i.e. simultaneous intra and inter-protein interactions). Therefore, tendency to form simultaneous intra and inter-protein interaction is elicited by almost all the residue types which show tendency to occur in protein-protein interfaces. Interestingly, these residue types include hydrophobic residue (like Leu, Phe, Trp and Met) and also polar residues (like Asp, Glu, His and Arg). It is also interesting to note that despite Gln and Lys being long side chains, they do not show high preference for simultaneous inter- and intra-protein interactions though they show propensity greater than 1 for inter-protein interactions. In contrast, Glu and Arg show high tendency for both being at the interface, as well as simultaneous intra- and inter- protein interactions.

The residue types low propensity (< 1) to occur in protein-protein interface also show low propensity (< 1) to form simultaneous intra- and inter-protein interactions. Therefore, formation of simultaneous intra- and inter-protein interactions is a general feature of interfacial residues almost irrespective of residue types.

**Fig. 2:**
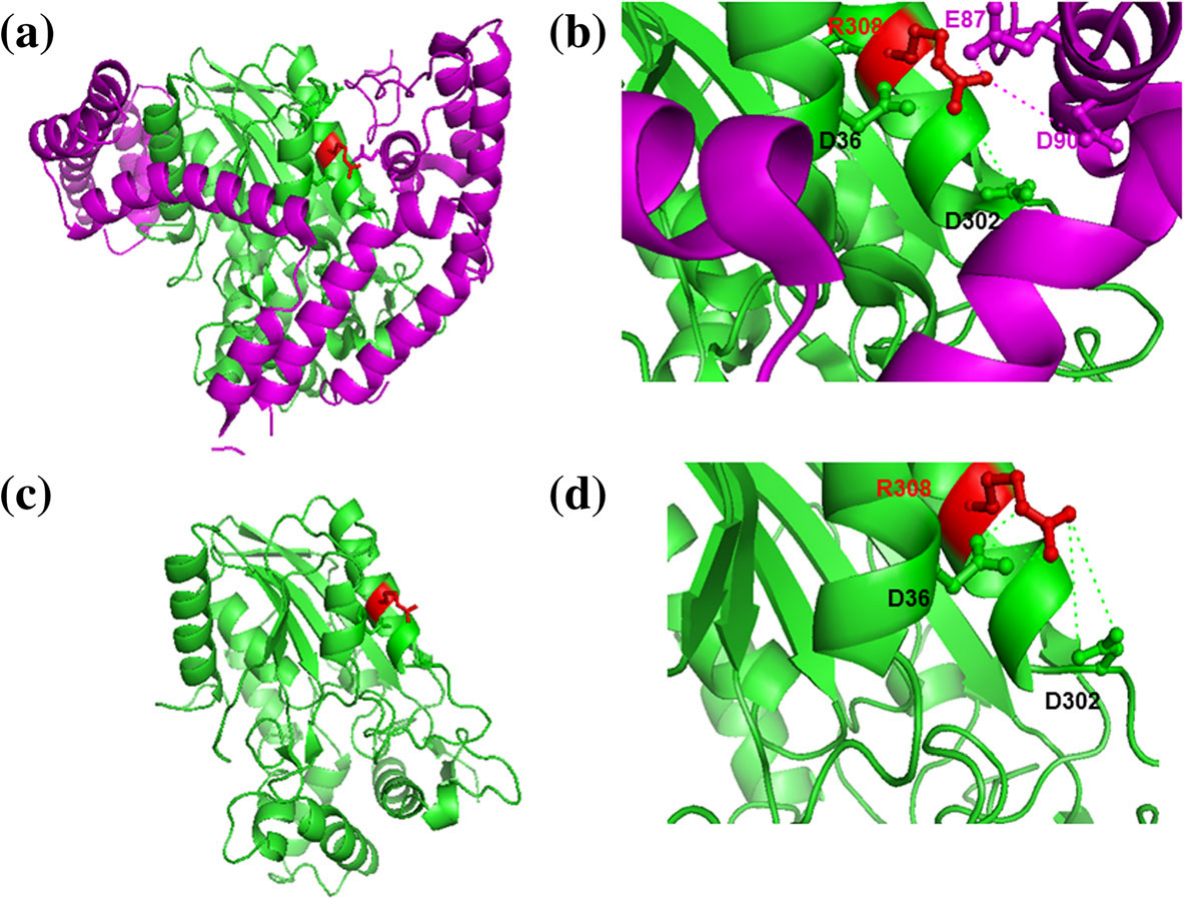
An example of protein-protein interactions with some of the interface residues forming simultaneous inter and intra protein interactions. This example corresponds to a bacterial lipase in complex with cognate foldase. Complex form (PDB code: 2es4) (a, b) and unbound form of lipase (PDB code: 1cvl) (c, d). (**a**) An interface residue Arg 308 (shown in red colour) of lipase (backbone shown in green) is engaged in bifurcated interactions within and across proteins. B chain corresponds to cognate foldase and is shown in pink colour. (**b**) Zoom-up of this interface region. Side chains of interacting residues across chain are shown in pink colour. Side chains of interacting residues within lipase are shown in green colour and from foldase in pink colour. Interactions are marked in pink and green dashes, respectively. Interacting residue names and numbers are marked. (**c**) Uncomplexed form of lipase. Side chains of residue, Arg 308, and residues within interacting distance within lipase are shown, as in (a), in red and green colour, respectively. (**d**) Zoom-up of the same as in (b) but for the uncomplexed form. Interactions within lipase are remarkably well-preserved between the uncomplexed and complexed forms of lipase. This interaction is augmented by two charged-residue interactions (Glu 87 and Asp 90) which are with the foldase. This figure and Fig. 3 were generated using PyMOL [16]

**Fig. 3:**
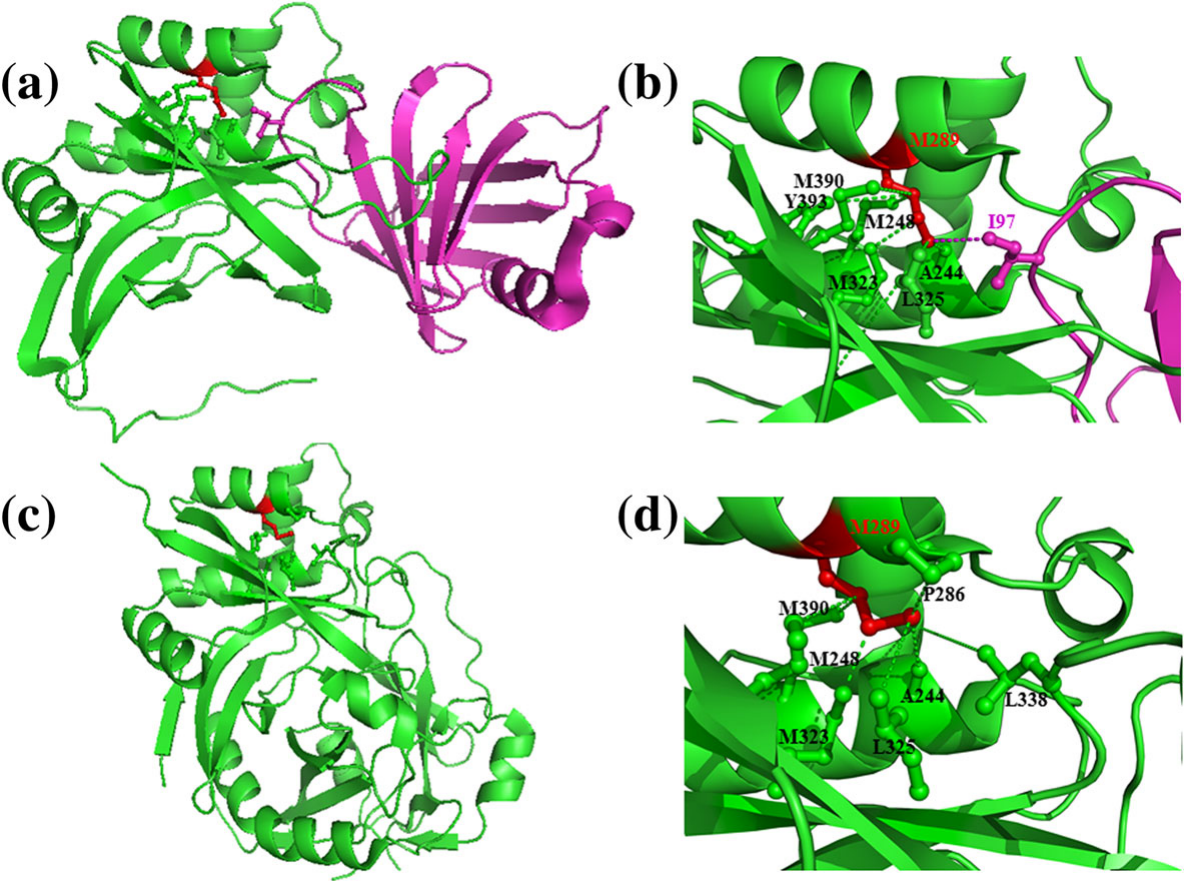
same as in Fig. 2, but for (**a**) Staphostatin (inhibitor) -staphopain (cysteine proteinase) complex (PDB code: 1pxv) and (**c**) prostaphopain B structure which is the precursor form of staphopain proteinase (PDB code 1x9y). Cysteine proteinase is shown in green colour and the inhibitor in pink. (**b**) and (**d**) show the interactions in the zoomed up form corresponding to an interface residue, Met 289. The side chain of Met 289 is shown in red, those of intra-protein interacting residues in green and those of inter-protein interacting residues in pink, as in Fig. 2. Most of the intra-protein interactions of one interface residue, Met 289 involved in bifurcated interactions, is shown to be retained as in the uncomplexed precursor form. Interestingly, one of the key intra-protein interactions (Met 289 to Leu 338), observed in the precursor form is replaced by two inter-protein interactions (Met 289 of protease to Ile 97 of the inhibitor)

Figures 2 and 3 show examples of simultaneous interactions involving interface residues, Arginine and Methionine, engaged in bifurcated interactions in protein-protein complexes corresponding to PDB codes 2es4 and 1pxv, respectively. Arginine, as shown in the example, forms intra- and inter-protein interactions with negatively charged residues (Aspartic and Glutamic acid). Methionine residue, as shown in the second example, forms hydrophobic interactions with neighbouring residues (Methionine, Proline, Alanine and Leucine/Isoleucine). Interestingly, residues involved in most of the inter-protein interactions are engaged in intra-protein interactions in the uncomplexed form and vice versa. For instance, Met-Leu interaction, within protein in the uncomplexed form, is replaced by Met-Ile interaction in the protein-protein complex (Fig. 3).

### Energy contributions of residue types to form intra and inter-protein interactions

**Fig. 4:**
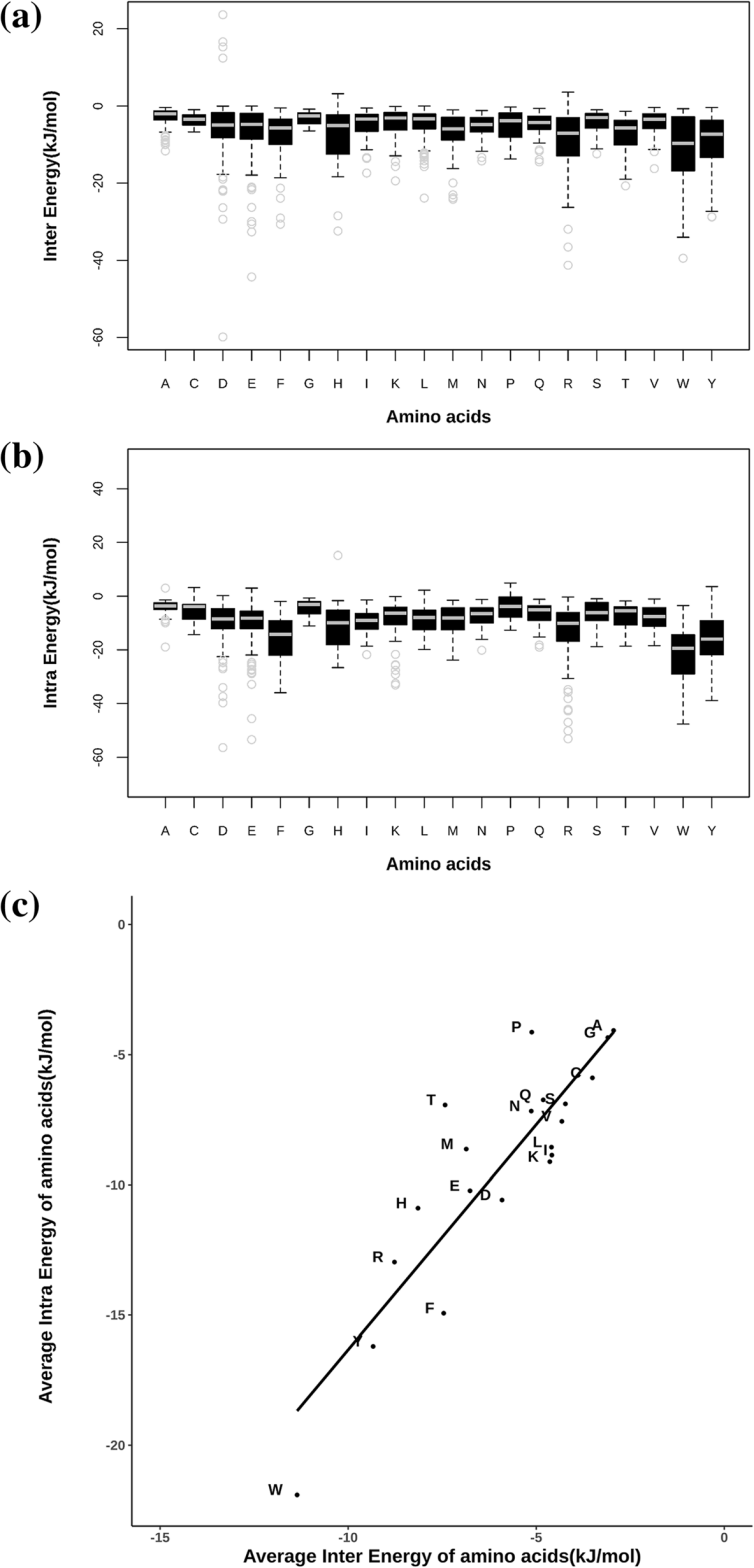
The distribution of PPCheck energies for each of the 20 amino acid types shown as box and whisker plots. Amino acids are indicated in single letter code. (a) inter-protein interactions and (b) intra-protein interactions. Least-squares fit line is shown. Correlation-coefficient is 0.88. (c) correlation between average PPCheck intra-protein and PPCheck inter-protein energies for the 20 amino acid types

Energy values associated with interactions made by the sidechain atoms of interfacial residues, in the protein-protein complex structure, with surrounding atoms were calculated using PPCheck [14]. The total energy of interaction associated with the sidechain of every interfacial residue is represented as the sum of energy associated with inter- and intra-protein interactions. Distributions of inter- and intra-protein interaction energies, spanning the entire dataset but partitioned into each of the 20 residue types, are shown in Fig. 4a and b, respectively. Overall span of energy values with mean value are shown for each of the residue types.

From Fig. 4a, it is evident that the residue types associated with least inter-protein interaction energy values are Trp, Tyr, His and Arg. From Fig. 4b, it can be noticed that the interfacial residue types associated with least intra-protein interaction energy values are Trp, Tyr, Phe, His and Arg. Figure 4c shows that the inter- and intra-protein interaction energy values for each of the 20 residue types are reasonably well-correlated. As most residue types that are associated with lowest intra- and inter-protein interaction energy values are common, it could be inferred that a residue type preferred at the protein-protein interface, overall, contributes substantial energy of stabilization both through inter- and intra-protein interactions.

**Fig. 5:**
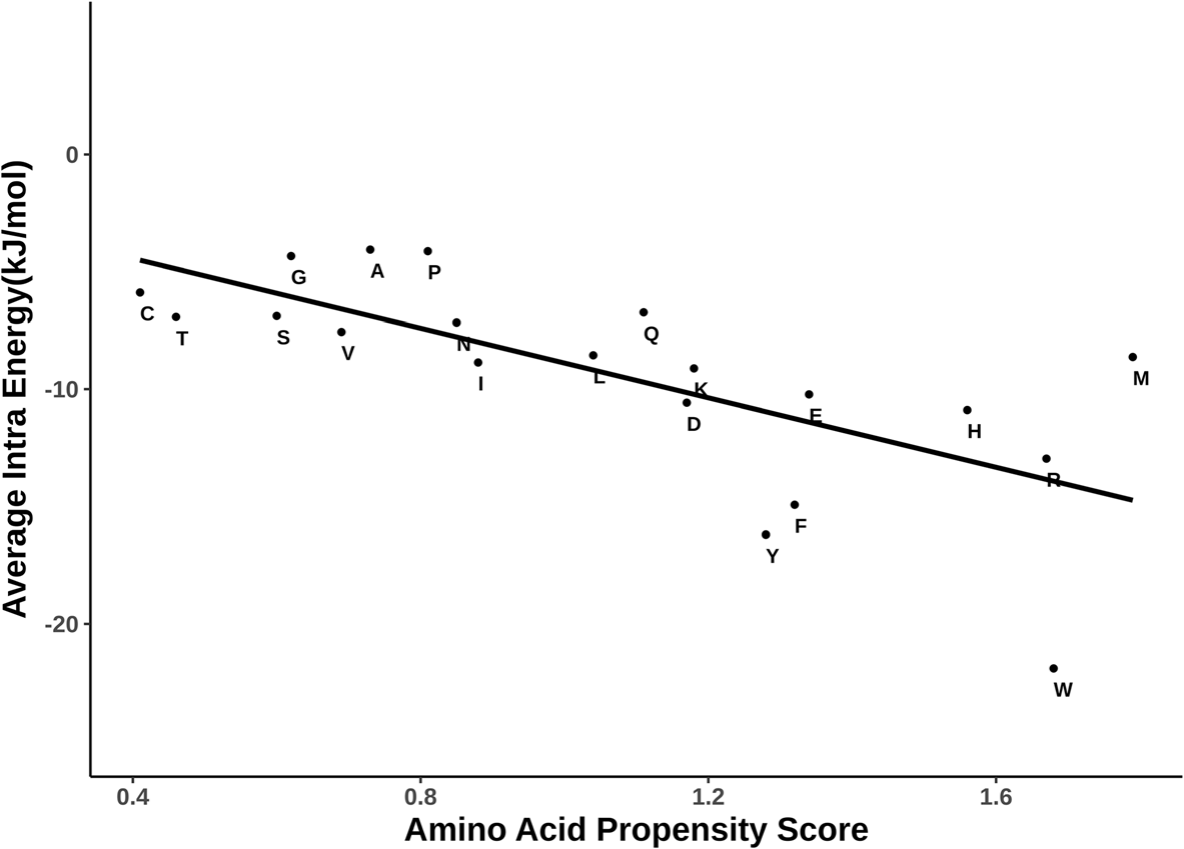
Comparison of 20 amino acids for amino acid propensity score (as measured through PIC program) versus average intra-protein energies (as measured by PPCHECK). Amino acids are indicated in single letter code. Least-squares fit line is shown. Correlation-coefficient is − 0.7

This learning is strengthened further by the scatter plot shown in Fig. 5 in which propensity of 20 residue types to occur in the interface is shown along X-axis and mean intra-protein interaction energy values for each of the 20 residue types along the Y-axis. Reasonably good correlation between the two distributions confirms that residue types preferred at the protein-protein interfaces generally render stability to the complex through intra-protein interactions, aside from inter-protein interactions.

### Contribution of inter and intra-protein interaction energies by the interfacial residues

While it is evident that residue types which prefer to be at protein-protein interfaces are also involved in intra-protein interactions, it is not clear how far individual interfacial residues contribute towards inter and intra-protein interaction energies.

**Fig. 6:**
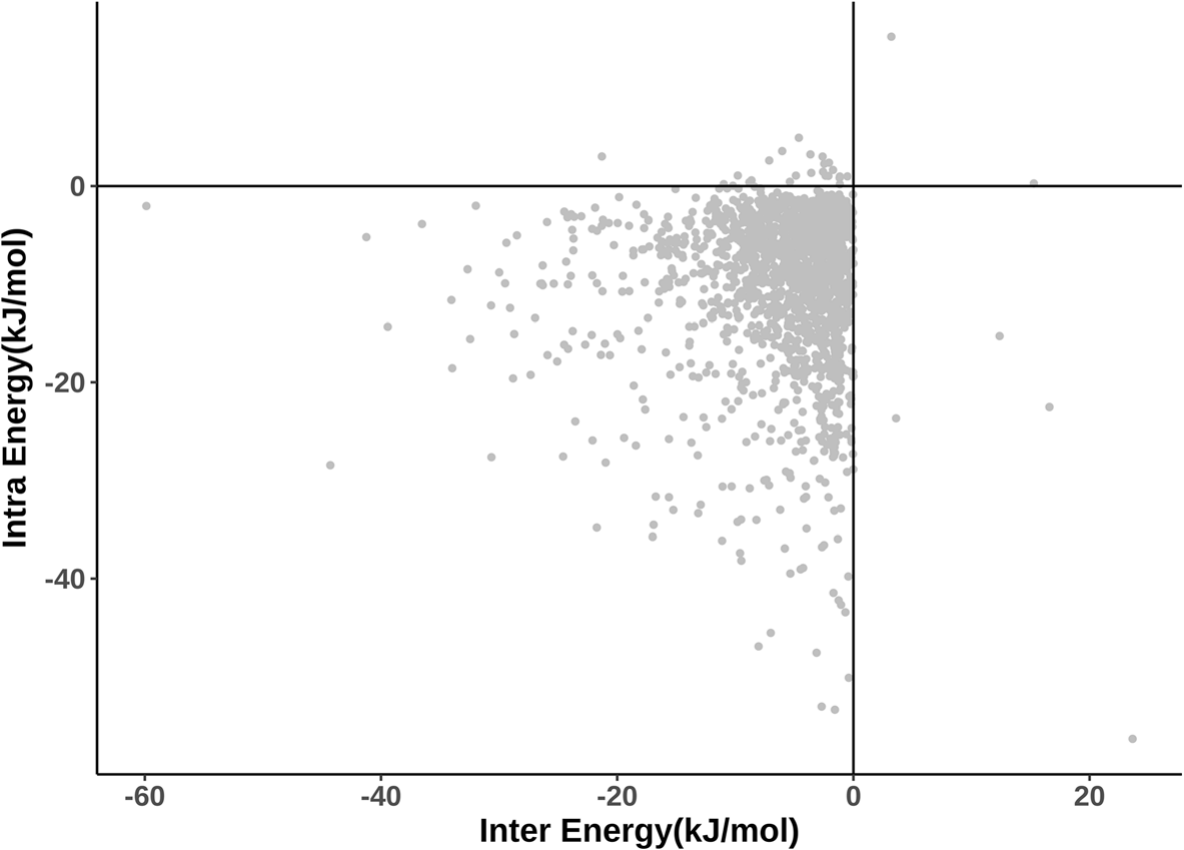
Scatter plot of PPCheck intra-protein interaction energy and inter-protein interaction energy for all 2137 interface residues in the entire dataset

Figure 6 shows a scatter plot between intra-protein interaction energy and inter-protein interaction energy for the 2137 protein-protein interfacial residues in the dataset. It can be noticed that intra- and inter-protein interaction energy values are quite similar for many residues. Therefore, most of the interfacial residues in the dataset contribute intra-protein interaction energy, almost as much as their energy contribution through inter-protein interactions. Considering a vertical streak of points close to the Y-axis, it is clear that there are a number of residues with energy contributions through intra-protein interactions much more than the contribution through inter-protein interactions. Indeed, the number of points with opposite trend i.e., more substantial contribution through inter-protein interactions than through intra-protein interactions is clearly much smaller, suggesting that intra-protein interactions contribute highly towards the stability of the protein-protein complexes.

#### Illustrative examples

Interestingly, in the examples of protein-protein complex structures, illustrated in Figs. 2 and 3, the interactions that are formed with residues within the protein are largely observed in the protein-unbound form. Such a trend is observed in a majority of instances in the dataset. Only four complexes show low retention of intra-protein interactions between complexed and uncomplexed forms (PDB codes 1f3v, 1nrj, 2fcw and 2vn5 of the complexed fom). These are reported to undergo huge structural changes upon complex formation. Additional file 4: Table S3 provides the list of interacting residues in the bound and unbound forms for those interface residues which are involved in bifurcated interactions. Additional file 5: Table S4 lists the percentage of intra-protein interactions of interface residues (engaged in bifurcated interactions) observed in the uncomplexed form as well. If one considers 41 out of 45 transient complexes in the current analysis, except the four cases mentioned above, the average percentage is 82.7%. This suggests that the microenvironment for interface residues, to form bifurcated interactions remain preformed and stable even before complexation with the partner protein. It further suggests that certain residues in such transient protein-protein complexes do not undergo huge structural changes at the interface regions between unbound and bound forms. This is consistent with the observations made earlier [9].

## Conclusions

In this paper, we demonstrate and highlight the fact that residues at the protein-protein interfaces contribute substantially to the stability of the complex, not only by inter-protein interactions, but also by intra-protein interactions. Clearly, the shape, conformation, chemical nature and nature and extent of dynamics associated with the interface in a protein are quite important in conferring stability and specificity of protein-protein complexes [15]. Therefore, side chains of protein-protein interfacial residues play double role - by directly contributing to the stability of the complex by interaction with the binding protein and also by interactions with proximal atoms in the protein which accommodates the residue concerned.

We also show that intra-protein interactions are a general feature of almost all the interfacial residues. The nature and extent of energy contribution in such “self-stabilizing” interactions differ between interfacial residues. The energy contribution from intra-protein interactions is shown to be quite substantial. Residue types with good propensity for simultaneous intra and inter protein interactions include hydrophobic residues Leu, Phe, Trp and Met and also polar residues Asp, Glu, His and Arg. Therefore, the simultaneous intra and inter-protein interactions include various kinds, such as interactions between hydrophobic groups and hydrogen bonds.

The learning from this work encourages one to consider intra-protein interactions by the interfacial residues, apart from inter-protein interactions, while designing site-directed mutants, tinkering the stability/specificity of a protein-protein complex and in the de novo design of protein-protein complexes.

## Reviewer’s comments

### Reviewer 1: Arumay pal

Comment:

In this work, Jayashree S. et al. analyzed the residue interactions that occur in their dataset of 45 high-resolution transient protein-protein binary interfaces using in-house programmes. They defined the interaction of interface residues into two categories- ‘solely inter-protein’ and ‘bifurcated’ (simultaneous intra and inter-protein interactions). The major findings include- i) 75% of the interface residues are of bifurcated type, ii) Bifurcated residue propensities are similar to residue propensities in PP-interfaces where aromatic, hydrophobic and charged side chains (except Lys) occur more compared to polar side chains, and iii) the energy contribution of the interface residues are higher for intra-protein interactions than inter-protein interactions in general, though the later can be equal to or even more than the former in cases. The findings can be useful for the better understanding of the structural aspects of transient PPI, which in turn can be useful for PPI design. The write-up, standard of English and data presentation is satisfactory. I would like to recommend the work suitable to be published in Biology Direct.

*Response:*

*We thank the reviewer for a nice summary of our work and for highlighting positive aspects in our work.*

Comment:

Following minor issues are needed to be addressed before publishing. I would like to highlight a few general comments as well. Minor issues: 1) The definition of an interface residue is not clear. 2) Authors found that 75% of the interface residues are of bifurcated type. While this is the overall statistic, it will be interesting to see the distribution of the percentage of such residues in each interface. For example, an interface full of (> 90%, say) bifurcated residues should be more suitable to design an inhibitory peptide. 3) Comment about the rest 25% interface residues and their propensities. 4) The dataset can be divided into two parts depending on the extent of conformational changes (low and high) upon binding. Will be interesting to see if there are differences in trends for the two sets, as found in the case of 4 complexes that undergo large conformational changes. 5) Methods (Identification and categorization of residue-residue interactions) – ‘It is possible that the same residue pair could be listed in more than one type if there are simultaneous van der Waals and hydrogen bond interactions etc. In such instances, interaction at the residue pair was counted only once.’- which interaction is given preference and why? 6) Fig. 1 – A regression line can be added, the correlation coefficient can be shown, and two dotted lines, horizontal and vertical, along the 1.0 values can be drawn for better clarity. 7) Figs. 2 and 3 – I would put the protein in lighter shades (eg. Light green, light violet etc.). 8) Fig. 4C – All fonts must be increased. A regression line can be added showing the correlation coefficient. 9) Fig. 5 - A regression line can be added showing the correlation coefficient.

*Response:*

*1) We have defined the interface residues better in the revised manuscript (Section 2 under Methods).*

*2) A new supplementary table (*Additional file 2*: Table S2) has been included with % of interfacial residues involved in bifurcated interactions for every protein chain used in the data set. A histogram showing the distribution is also included (*Additional file 3*: Fig. S1 of the revised manuscript).*

*3) We have now commented on the 25% of the cases in the revised manuscript (towards the end of first subsection under Results and Discussion.*

*4) We thank the reviewer for this nice suggestion. However, we are facing two problems in performing this analysis at the present time: (1) Data set for this analysis require 3-D structures of both protein-protein complex and 3-D structures of proteins involved in their uncomplexed forms. Though we have used such a data set in our analysis, the number of protein-protein complexes which show substantial change in conformation upon complexation is too few to show a clear pattern compared to the complexes which do not change the structure significantly upon binding. (2) In some of the complexes of two proteins, one of the proteins undergo substantial conformational change upon binding, while the other does not show much conformational change. Such complexes cause difficulty in classifying them into “low conformational change complex” or “high conformational change complex”.*

*We feel that this interesting project should be carried out when the large dataset could be formed, with clarity in the definition of protein-protein complexes with low and high conformational change.*

*5) We are sorry that these statements look misleading. We have now rewritten this part to give the correct message. Basically, it is possible that the same residue pair could be listed in more than one type if there are simultaneous van der Waals and hydrogen bond interactions* etc. *In such instances, the pair with interacting residues was counted only once, though the number of interactions between the same two residues could be more than one.*

*6) Thanks for this suggestion.* Figure 1 *has been modified to show the least squares line and vertical & horizontal lines at propensity value of 1. Correlation-coefficient value is provided in the legend to the figure.*

*7)* Figs. 2 and 3 *are protein structural pictures in white background. When we tried the suggested colours, they were not shown well in the figure. Therefore, we prefer to leave these figures unchanged. However, we are open for any suggestion that will improve the figures.*

*8) All the suggested changes in* Fig. 4C *have been made (increase in font size and least squares line). .*

*Correlation-coefficient value is provided in the legend to the figure.*

*9) All the suggested changes in* Fig. 5 *have been made. Correlation-coefficient value is provided in the legend.*

General comments: 1) Conservation of residues involved in ‘solely inter-protein’ vs. ‘bifurcated’ interactions can be checked. 2) An abstract graphics could be used to describe the concept and the major findings easily. 3) Since Fig. 4C and Fig. 6 are coupled, they could be placed together.

*Response:*

*Concerning point 1 above, as can be seen from the section on propensity calculations, the propensities of residue types to form simultaneous intra- and inter-protein interactions are very close to the general propensities of residue types to occur in protein-protein interfaces in general. It is well known in the literature that protein-protein interfacial residues are reasonably well conserved (Works of Janet Thornton, Pinak Chakrabarti, Joel Janin and many others). Therefore, it is only expected that the residues which form bifurcated interactions and those which are involved in inter-protein interactions are reasonably well conserved.*

*Regarding graphical abstract (point 2 above), we will be happy to provide one if journal requires it.*

*Regarding coupling* Fig. 4C and 6 *in a single figure, we see the point of the reviewer. But, it will compromise on the discussion of* Fig. 4 *as 4C is discussed in relation to 4B and 4A. However, if it is strongly felt that these figures should be combined into one, we will do our best in rewriting those sections not to affect the readability of the paper.*

Comment:

Typos: 1. Methods, line 51 - categorization 2. Line 53 - A fullstop after [12] 3. Results and discussion, line 39 - “In the complex structures,...”

*Response:*

*Thanks. All these typos have been corrected in the revised manuscript.*

### Reviewer 2: Mallur Madhusudhan

Comment:

This manuscript attempts to categorise interface residues according to whether they mediate only inter-chain interactions or whether they participate in both inter- and intra-chain interactions. Propensity values for all 20 amino acids are drawn on this basis. The results could however be interpreted as being a trivial outcome of amino acid size. One potentially interesting aspect of this study is the observation that the interaction environment of the residues in the uncompleted and complexed forms are similar. This idea is however not explored in detail.

*Response:*

*The main take-home message of our work is that the most of the interfacial residues in a transient protein-protein complex are also involved in intra-protein interactions. To the best of our knowledge, this has not been shown previously using a systematic analysis. Secondly, it is our belief that this is an important result as this is likely to have important implications in engineering protein-protein interactions, in the design of inhibitors of protein-protein complexation* etc.

*Addressing the point of size dependency of residue types on the tendency to form bifurcated interactions, it must be noted from the section on the propensity calculations that both long/bulky sidechains (*e.g.*, Arg and Phe) and not so long sidechains (*e.g.*, Asp and Leu) show tendency to form bifurcated interactions. Based on such observations, we think that there is no clear size dependency on the tendency to form simultaneous intra- and inter-protein interactions.*

*Concerning the point made by the reviewer on our results of comparison of complexed and uncomplexed structural forms of proteins, our main message is that most of the residues involved in simultaneous intra- and inter-protein interactions in the complexed form are also involved in intra-protein interactions in the uncomplexed form. We believe that we have presented concrete data and discussed it in the manuscript in sufficient detail.*

Comment:

The manuscript by Srinivasan and coworkers attempts to decipher the roles of residues on protein-protein interfaces, specifically of interfaces involved in transient interactions. The authors have bifurcated residues at the interface into two types – those that make interactions only with the interactor (inter-) and those that make interactions with the interactor and residues of their own protein (intra/inter-). The main results of the finding are that a large number of residues belong to the latter category and there is seemingly no preference of amino acid type in defining one type over another. More interestingly, the authors point out that when they analyze the protein structures in the uncomplexed form, the interaction of intra/inter- residue tend to conserve their interaction environment. While the manuscript is clearly written several of the analysis are not convincing.

*Response:*

*We thank the reviewer for the comments and constructive criticisms on our work. We find it helpful in strengthening our work. We are providing our point-by-point response below.*

Comment:

The authors should address the following criticisms and comments 1. Why have the authors only considered transient complexes? This choice has not been justified. Presumably, this inter and intra/inter property of residues would be a feature of all protein-protein interactions (as implied in the opening section of the manuscript). Why then were transient interactions selected? Is there reason to believe that the behavior of interface residue in transient interactions are different than those mediating obligate interactions?

*Response:*

*Questions we addressed in our work and the analysis we carried out requires availability of experimentally determined 3-D structures protein-protein complexes and structures of same proteins in the uncomplexed form. These conditions are necessarily met only by the transient protein-protein complexes as the permanent complexes, by definition, are not stable in isolation (uncomplexed form) and therefore cannot be crystallized in isolation.*

*Further, as also commented by the other reviewer of our paper, we believe performing this analysis on the transient complexes would be more useful, especially in the context of design of small molecules that target protein-protein interfaces. We believe targeting the interface of permanent complexes is less attractive and more challenging as the chances of success appear very small.*

*Having said this, suggestion of the reviewer is well taken – in a separate and explicit project, we will analyze the interfaces of permanent complexes for simultaneous intra- and inter-protein interactions.*

Comment:

2. The authors have in different parts of the manuscript expressed surprise at the proportion of intra/inter- interacting residues. They also point out there appears to be no residue-type preference to be an intra or intra/inter- residue. This reviewer has an alternate explanation, which is apparent from Figs. 1 and 5 (and Fig. 4c?) – It is reasonable to expect small amino acids at the interface to participate in inter-chain interactions while larger residues by the virtue of having more atoms in the side chain are more likely to participate in intra/inter-chain interactions. The data presented in Figs. 1 and 5 (and 4c?) can be interpreted more simply – They cluster small and big residues at the opposite extremes. It is no surprise then that Cys, Thr, Ser, ala, Gly are predominantly of the inter- type while Trp, met, Arg, etc. have a strong presence in the intra/inter- type. Is this not a simpler way of analyzing the data? This also means that there is no real need to compute energies of interactions. The explanation based on size given above may not account for the behavior of Lys and Gln – Which appear to be at the border of the inter- and intra/inter- divide. Perhaps this is because of insufficient data? Would taking a larger dataset (including obligate interactions) for analysis have given a clearer picture?

*Response:*

*We thank the reviewer for an alternate interpretation of our data. As mentioned above, we find both long (Arg, Phe etc) and short sidechains (Asp, Leu etc) in the dataset of interfacial residues which are involved in simultaneous intra and inter-protein interactions. Reviewer has also noted the behaviour of Lys and Gln as deviant from the hypothesis on size dependency.*

*But it must be noted that the main new conclusion we report in our paper, that most of the interfacial residues are involved in bifurcated interactions, is strongly supported by our data analysis irrespective of the interpretations on the nature of sidechains involved.*

*We feel that energy calculations are necessary in order to reach an understanding on the comparative strengths of intra- and inter-protein interactions.*

Comment:

3. The one interesting observation of this study is that interface residue in uncomplexed monomers tend to preserve the interaction environment when they are part of a complex. However, the data shown in support of this claim need to be more substantive. This is a crucial part of the analysis and is likely to be of some importance to researchers in the field. The authors should concentrate on getting more concrete evidence of this fact. In their analysis 78% (the authors report this as 83% by discounting 4 poorly performing cases) of the interactions are common to the uncomplexed and complexed cases (Additional file 4: Table S3). This appears to be an interesting result and warrants investigation.

*Response:*

*We thank the reviewer for positive comments on our work on comparison of complexed and free forms of proteins. By the sheer high proportion (over 75%) of common residues involved in intra-protein interactions in complexed and free forms, we believe that there is no doubt on our conclusion. We would set out to address fresh useful questions on this aspect in a separate project. We thank the reviewer for this suggestion.*

Comment:

4. An important control is missing in this study. The authors claim that interface residues contribute significantly to intra-protein interactions. Their interpretation is that this strengthens the protein-protein interaction. Another way of looking at it would be that these residues are contributing to the integrity of the interface structure. The authors should contract this with other residues on the surface that are not known to be a part of any interface and how these residues interact with other residues of the same protein.

*Response:*

*We agree with the reviewer that intra-protein interactions by the interfacial residues contribute to the integrity of the conformation of the interfacial region. In fact, we did not mean that intra-protein interactions directly contribute to strengthening protein-protein interaction. We have ensured that, in the revised manuscript, we did not give the impression of intra-protein interactions contributing directly to the strength of protein-protein association.*

Comment:

Minor Points: 1. The manuscript has many places where it would help if the analysis were quantitative instead of qualitative. Instances of this could be found on lines such as – “..residues involved in most of the inter…”; “..20 residue types are reasonably well correlated with ..”; “Reasonably good correlation …”; etc. 2. It would be better to colour the atoms in Figs. 2 and 3 by hetero-atom. This would make the representation clearer. 3. Figure 4C is of very poor quality and undecipherable. 4. Figure 6 is uninformative (for the reasons explained above). 5. Some references need to be added (Chou-Fasman, PIC server).

*Response:*

*Regarding point 1 above, we have ensured that in the revised manuscript we provide quantitative information (in the text, supplementary Table or figure) to support the statements. Correlation coefficient values are now mentioned in the legends to appropriate figures.*

*On the point 2 above, we have ensured that the two proteins involved in various panels in* Figs. 2 and 3 *and interactions are clearly shown in distinct colours. There are no het atoms involved in these figures.*

*Regarding* Fig. 4 *(point 3 above), we have re-done the figure with better clarity.*

*On the point 4 above,* Fig. 6 *provides a comparison of intra-protein interaction energy and inter-protein interaction energy. This figure provides information on strengths of intra-protein and inter-protein interactions. Such a information is pertinent to our paper, as we report extensive involvement of interfacial residues in the intra-protein interaction as well. As* Fig. 6 *provides useful and relevant information we would like to retain this figure and associated discussion in our manuscript. But, we are open to removing this figure and associated discussion, if it is strongly felt that this figure should be deleted.*

*The references of Chou-Fasman and PIC are included.*

## Additional files

Additional file 1: **Table S1.** List of transient protein-protein complexes used in the analysis (shown in green colour). PDB code of the unbound form of protein 1 is shown in pink. Where available, the PDB code of the unbound form of the second subunit is also noted (as shown in blue colour). (XLSX 16 kb)

Additional file 2: **Table S2.** List of PDB chains and percentage interface residues involved in bifurcated (both intra- and inter-chain) interactions. The first column shows the PDB code as well as the interacting chains. (XLSX 13 kb)

Additional file 3: **Figure S1.** Percentage distribution of interfacial residues involved in bifurcated interactions in the protein-protein complex structures used in the current study. (TIFF 569 kb)

Additional file 4: **Table S3.** List of interacting residues in the bound and unbound forms for those interface residues which are involved in bifurcated interactions. (please also see Additional file 1: Table S1) (XLS 231 kb)

Additional file 5: **Table S4.** Number of intra-protein interactions involving interface residues. The number of intra-protein interactions are compared with those in the uncomplexed form. Common intra-protein interactions between complexed and uncomplexed forms are expressed as percentage. (XLS 26 kb)

## Abbreviations

PDB: Protein data bank
PIC: Protein Interactions Calculator
PPI: Protein-Protein Interactions
